# Sub-nanometer-scale mapping of crystal orientation and depth-dependent structure of dislocation cores in SrTiO_3_

**DOI:** 10.1038/s41467-023-35877-7

**Published:** 2023-01-11

**Authors:** Haozhi Sha, Yunpeng Ma, Guoping Cao, Jizhe Cui, Wenfeng Yang, Qian Li, Rong Yu

**Affiliations:** 1 School of Materials Science and Engineering, Tsinghua University, Beijing, 100084 China; 2 MOE Key Laboratory of Advanced Materials, Tsinghua University, Beijing, 100084 China; 3 State Key Laboratory of New Ceramics and Fine Processing, Tsinghua University, Beijing, 100084 China

**Keywords:** Structural properties, Imaging techniques

## Abstract

Defects in crystals play a fundamental role in modulating mechanical, electrical, luminescent, and magnetic behaviors of materials. However, accurate measurement of defect structures is hindered by symmetry breaking and the corresponding complex modifications in atomic configuration and/or crystal tilt at the defects. Here, we report the deep-sub-angstrom resolution imaging of dislocation cores via multislice electron ptychography with adaptive propagator, which allows sub-nanometer scale mapping of crystal tilt in the vicinity of dislocation cores and simultaneous recovery of depth-dependent atomic structure of dislocations. The realization of deep-sub-angstrom resolution and depth-dependent imaging of defects shows great potential in revealing microstructures and properties of real materials and devices.

## Abstract (web summary)

Accurate measurement of defect structures is hindered by complex atomic configuration and/or crystal tilt. Here, the authors realize sub-nanometer mapping of crystal tilt and deep-subangstrom resolution and depth-dependent imaging of dislocations.

## Introduction

With the broken translation periodicity and lowered local symmetry, defects in materials play host to various physical and chemical phenomena. For example, dislocations are tightly related to many mechanical and physical properties, such as plasticity^1,2^, semiconductor luminescence^3^, piezoelectricity^4,5^, and magnetism^6^. Among these phenomena, atomic configurations near dislocation cores are of vital importance and have attracted much interest^7–10^.

Concomitant with the development of aberration-corrected electron microscopy, spatial resolution has been pushed to sub-angstrom, offering opportunities for imaging defects at atomic scale^11–15^. However, atomic-scale images of dislocations obtained with conventional electron microscopy are two-dimensional (2D) projections along dislocation lines, which are flexible and fluctuating at finite temperature. For example, because of the flexibility of dislocations and the Peierls potential, both thermal fluctuation and stress can induce kinks^16–18^, making the dislocation segments span several Peierls valleys and deviate from an ideal line. The structures of kinks take key roles in dislocation movement^19–21^. Remarkable efforts have been made to improve the resolution along dislocation lines to take advantage of aberration correction^22^. Larger convergence angle can be used to do depth sectioning^23–25^. Also, electron tomography has realized high-resolution imaging in 3D^26,27^. However, these methods mostly rely on high-angle annular dark-field (HAADF) image which is insensitive to light atoms like oxygen. Dynamic diffraction (e.g., the channeling effect) also degrades the accuracy of structure measurements.

Measurements of dislocation structures also suffer from the misorientation between the incident electron beam and the zone axis of crystalline materials. This effect can hardly be avoided because the strain field associated with dislocations may induce continuous lattice tilt, which reduces the measurement accuracy of atomic positions and related properties, like polar displacements in ferroelectric materials.

Ptychography is a real space phase-retrieval method which uses series of coherent diffraction patterns (the so-called four-dimensional (4D) datasets) to decode the sample potential from electron waves^28–31^. Utilizing iterative algorithms^32–38^, electron ptychography has reached deep sub-angstrom resolution in both 2D^39^ and bulk materials^40^. Capable of imaging both heavy and light atoms^41^, it becomes a powerful tool to investigate oxide materials. Very recently, the adaptive-propagator (APP) method^42^ was developed in the frame of multislice electron ptychography^34,43^ to remove the misorientation effect. It suggests possible application of deep-sub-angstrom resolution ptychographic imaging in real materials with intrinsic variation of crystal orientation around defects like dislocations and interfaces.

**Fig. 1:**
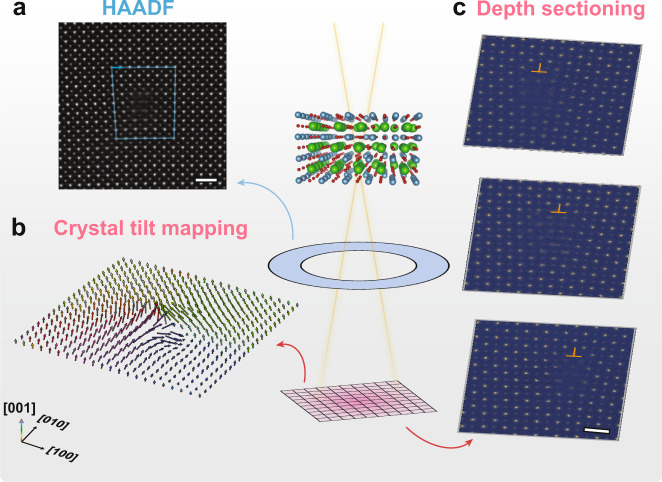
Schematics of the APP method for lattice tilt mapping and depth sectioning. **a** Wiener filtered HAADF image of a dislocation in SrTiO_3_. Scale bar, 1 nm. **b** 2D mapping of crystal tilt, namely the orientation of [001] direction of SrTiO_3_. **c** Depth sectioning of dislocation structure. Scale bar, 5 Å.

In this work, we report the deep-sub-angstrom resolution imaging of depth-dependent structure of dislocations and sub-nanometer mapping of crystal tilt in free-standing SrTiO_3_ films (Fig. 1). Transverse shift of the dislocations is revealed with the identification of kinks and is correlated with the tilt and strain in the films. The polarization displacements at different depth of the specimen are also obtained.

## Results

### Mapping of dislocation-induced crystal tilt

The forward model in iterative multislice ptychography algorithms is similar to the process of multislice simulation^44,45^. In the forward model of APP method, wave propagation between slices is described with Fresnel propagator, which is written as:

1 $$p\left({{{{{\boldsymbol{k}}}}}}{{{{{\rm{;}}}}}}\triangle z,{{{{{\boldsymbol{\theta }}}}}}\right)={{\exp }}\left[-i\pi \triangle z\left(\lambda {\left|{{{{{\boldsymbol{k}}}}}}\right|}^{2}-2{k}_{x}{{\tan }}{\theta }_{x}-2{k}_{y}{{\tan }}{\theta }_{y}\right)\right]\,,$$

where ***k*** is the 2D spatial frequency with *x* and *y* components (*k*_*x*_*, k*_*y*_), $\triangle z$ is the slice thickness, *λ* is the electron wavelength and ***θ*** = (*θ*_*x*_*, θ*_*y*_) is the tilt angle between incident beam and the zone axis of the sample. Throughout ***θ*** is used to demonstrate lattice tilt. In order to consider the spatial variation of the direction of the zone axis, ***θ*** can be defined as a pair of real space functions ***θ***(**r**) **=** (*θ*_*x*_(**r**)*, θ*_*y*_(**r**)). The tilt angle at each probe position can be retrieved together with the transmission function using well-developed optimization methods^46,47^.

**Fig. 2:**
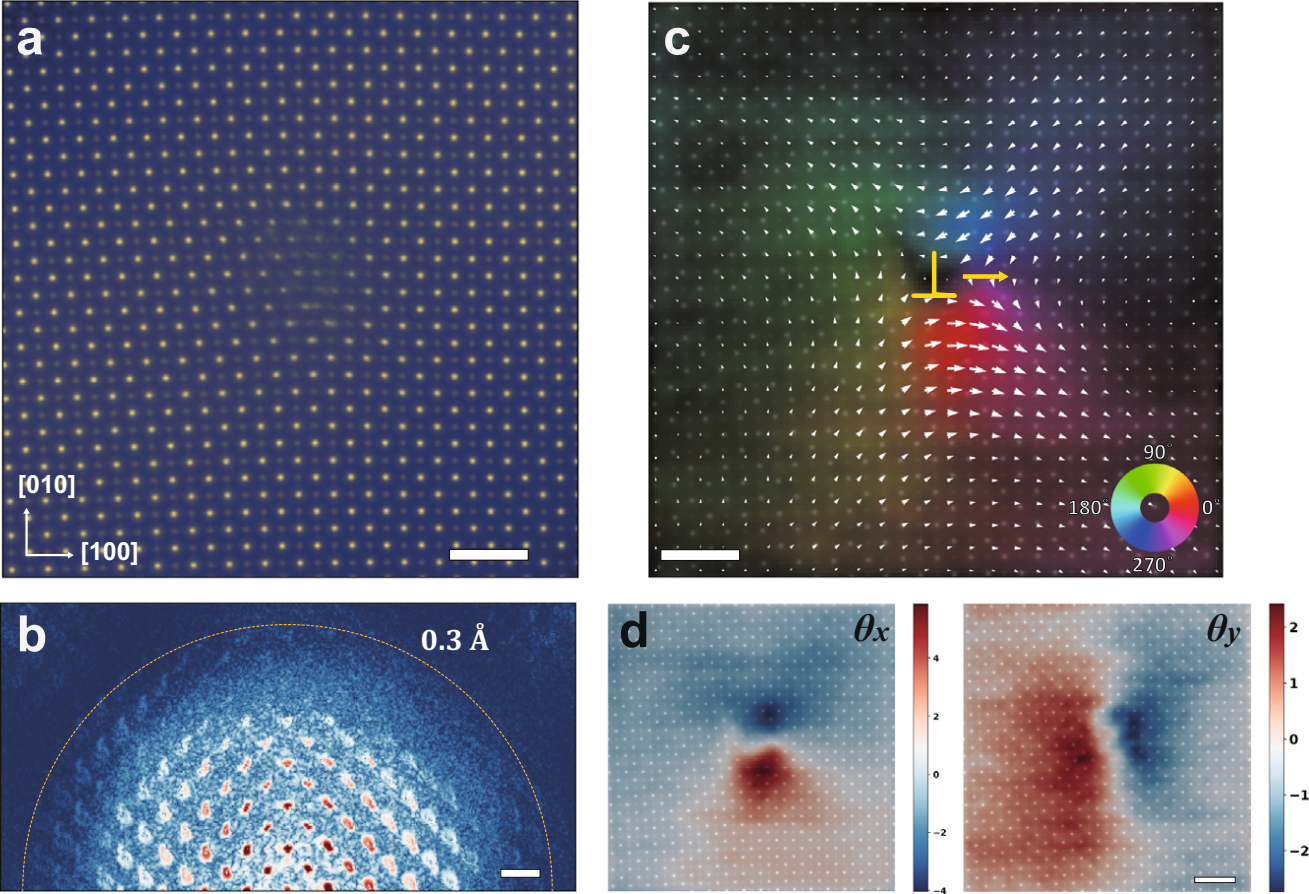
Structure imaging and orientation mapping of the dislocation in SrTiO_3_. **a** Total phase image summed over all the twenty slices recovered with APP. **b** Diffractogram of the total phase image shown in **a**. The yellow dashed line marks the information limit of 0.3 Å. Scale bar, 5 nm^−1^. **c** Crystal tilt mapping with total phase image superimposed on it. The white arrows stand for the in-plane projection of the [001] direction. The x and y components of the arrows are shown in **d**. The yellow arrow represents the transverse shift of the dislocation core. **d** Crystal tilts in [100] (left) and [010] (right) directions. Scale bars in **a**, **c** and **d** are 1 nm.

Free-standing films are highly flexible^48,49^ and are easily bended (shown with diffraction contrast in Supplementary Fig. 1a). In this study, the thickness of free-standing SrTiO_3_ film is about 15 nm. It was grown on the sacrificial Sr_2_CaAl_2_O_6_ layer of 18 nm thick on the SrTiO_3_ substrate. See the section Methods for more details about the thin-film preparation. A noticeable number of dislocations exist in the film, some of which are shown in Fig. 2, Supplementary Fig. 1 and Supplementary Fig. 2. Local stress field induced by the elastic deformation leads to a continuous crystal tilt, which distorts atom columns in the annular bright-field (ABF) image. In contrast, APP removes the misorientation effect and guarantees both heavy and light atomic columns to be clearly imaged (Fig. 2a). The diffractogram in Fig. 2b demonstrates a resolution beyond 0.3 Å. Dislocation induced crystal tilt is mapped and shown in Fig. 2c. The white arrows stand for the in-plane projection of the [001] directions in Fig. 1b, the x and y components of which are crystal tilts in [100] and [010] directions, respectively (Fig. 2d). In the spatial range of reconstruction, film bending deformation varies slowly, so the bending-induced orientation changes can be viewed as a constant. The mean value of crystal tilt in the region around the dislocation is subtracted from the tilt map, which includes bending-induced tilt and the overall sample mistilt (see Supplementary Note 1 for details). A larger field of view with three dislocations is shown in Supplementary Fig. 2a and the corresponding crystal tilt map is shown in Supplementary Fig. 2b. We notice that there may be a correspondence between the tilt map, the strain state, and the transverse shift of dislocations. In Supplementary Fig. 2c, we show a model that correlates well the observed tilt map with the transverse shift of the dislocations.

Based on the simulation in the Supplementary Note 2, the ideal resolution of crystal tilt under the experimental dose (4.4×10^5^ e/Å^2^) is about 0.8 nm, and the precision is about 0.4 mrad. However, for experimental dataset corresponding to results in Fig. 2, a low-pass filtering is performed to Supplementary Fig. 3a, b to reduce the noise in the measurements, leading to mapping shown in Fig. 2d. No filter is applied to results in Supplementary Fig. 2 and Supplementary Fig. 3e, f. The result inspires us to concern the local variation of crystal orientations caused by defects like dislocations, which may be linked to the changes of specific physical properties. Besides dislocations, strain fields in domain walls and interfaces of heterostructures may also cause crystal tilt. The ability of simultaneously retrieving both lattice tilt and atomic structures makes APP a powerful method to study defect-induced structure variations in materials.

### Kinks on the edge dislocation

For a long time, fine structure variations along the dislocation line, such as kinks and jogs are easily lost in the 2D projected images of samples under the ‘edge-on’ condition. Characterizing such 3D structures puts a high demand on the depth resolution of electron microscopy. The diffraction limit of depth resolution of HAADF and integrated differential phase contrast (iDPC) in scanning transmission electron microscopy (STEM) is 2λ/α^2^, where λ is the wavelength and α is the convergence semi-angle defined by the condenser aperture^50–52^. Previous work has observed kinks on a partial dislocation in silicon^8^, while the kinks on edge dislocations in bulk materials remain untapped at atomic scale. Benefiting from the state-of-the-art lateral resolution^39,40^ and a depth resolution beyond diffraction limit^40,53^, multislice ptychography can be used as a powerful tool to investigate 3D structure changes of dislocations.

**Fig. 3:**
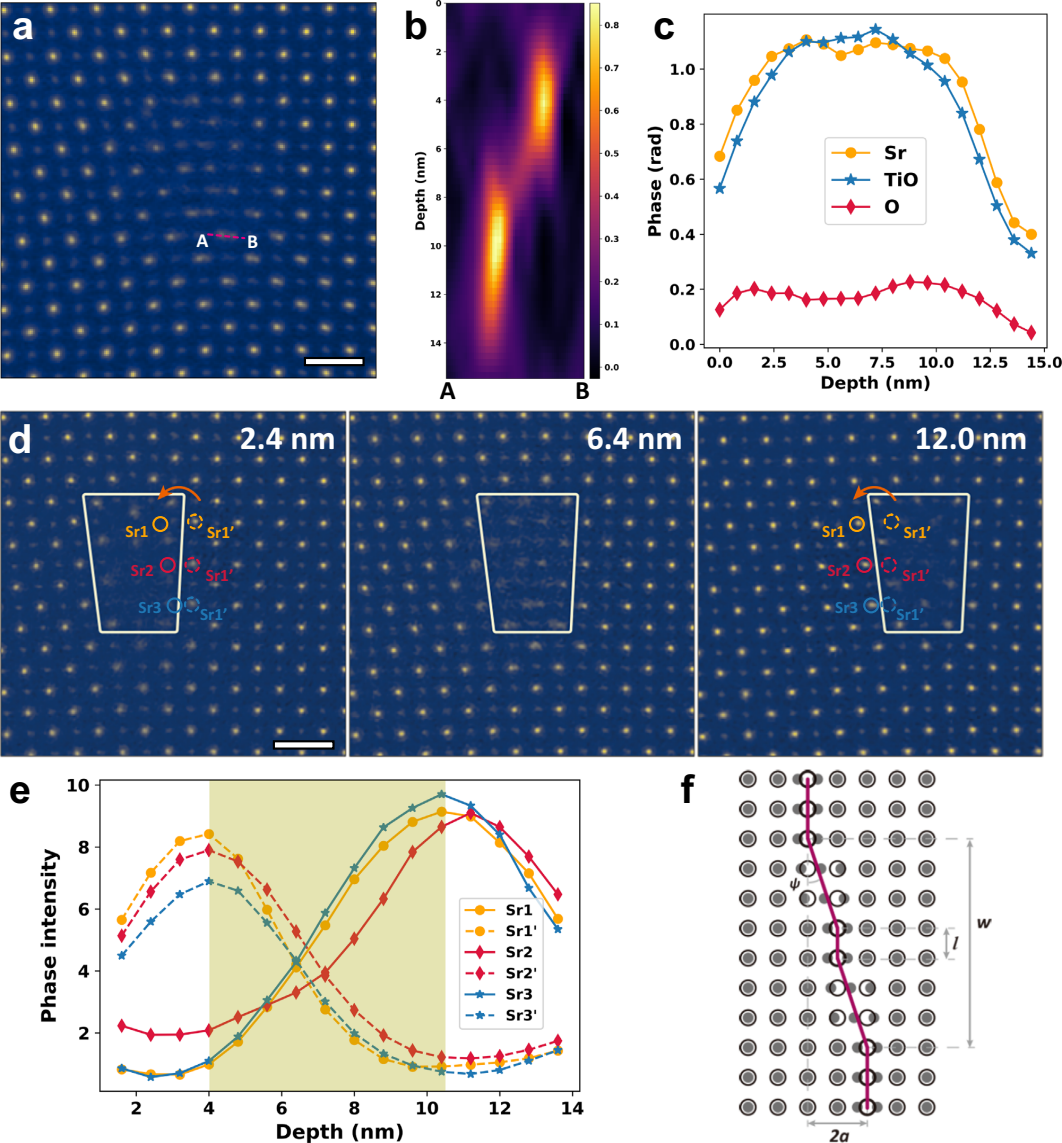
Three-dimensional visualization of the edge dislocation. **a** Total phase image of the edge dislocation. Scale bar, 5 Å. **b** Depth variation of the phase intensity across the split columns marked with A and B in **a**. **c** Depth profiles of Sr, TiO and O atomic columns. **d** Phase images at 2.4 nm, 6.4 nm and 12.0 nm in depth, respectively. Scale bars, 5 Å. **e** Phase profiles of the atomic columns marked in **d**. The green shading shows the region of the kink segment. **f** Schematics of kink configuration.

During ptychographic reconstruction, the sample is divided into 20 slices and each slice is 12 Å in thickness. The recovered phase images of all the slices are shown in Supplementary Movie 1 (depth sections of the region in Supplementary Fig. 2a are shown in Supplementary Movie 2). In the projected phase image (Fig. 3a), several atomic columns are split. The corresponding atomic columns are blurred in the HAADF and ABF images because of the limited lateral resolution (Supplementary Fig. 1c, d). Figure 3b shows the depth-dependent phase distribution across a pair of split columns marked with A and B in Fig. 3a. It clearly illustrates that the peak split is due to the lateral shift of the atomic column along the depth. Depth resolution can be estimated from the depth profiles shown in Fig. 3c, which is 3.8 nm for Sr using the full width at 80% of maximum (FW80M) of the fitted error function. This value is better than the value 6.5 nm limited by aperture and chromatic aberration (refer to Supplementary Note 3 for details of estimation of depth resolution).

As evident in Fig. 3d and Supplementary Movie 1, the edge dislocation is not a straight line but contains kinks spanning over two unit-cells. The three Sr columns marked in Fig. 3d shift from the dotted circles to the solid ones in depth, in accompany with the transverse shift of the dislocation core. Estimated from the phase profiles of these atomic columns (Fig. 3e), the total width *w* of the kink segment is about 6.5 nm as illustrated in Fig. 3f. Kink width is an important factor reflecting the magnitude of Peierls barrier and determining the elastic energy of kinks. More geometrical information of dislocations may be obtained if larger convergence semi-angle can be used in electron ptychography to improve the depth resolution of electron ptychography to sub-nanometer scale^53^.

We note that the integrated center-of-mass (iCOM) and its simplified form iDPC have been established as a depth-sectioning method with excellent depth resolution^52^. Here we compare the performance of multislice ptychography and iCOM/iDPC for depth-sectioning.

A structure model of SrTiO_3_ edge dislocation with a kink spanning over one unit-cell was built for the generation of a simulation dataset. The thickness of the model is 15 nm. The high voltage is 300 kV and convergence semi-angle is 25 mrad. Both the slice thickness for multislice ptychography and the defocus interval for iCOM are 1 nm. More simulation details can be found in the Method section.

**Fig. 4:**
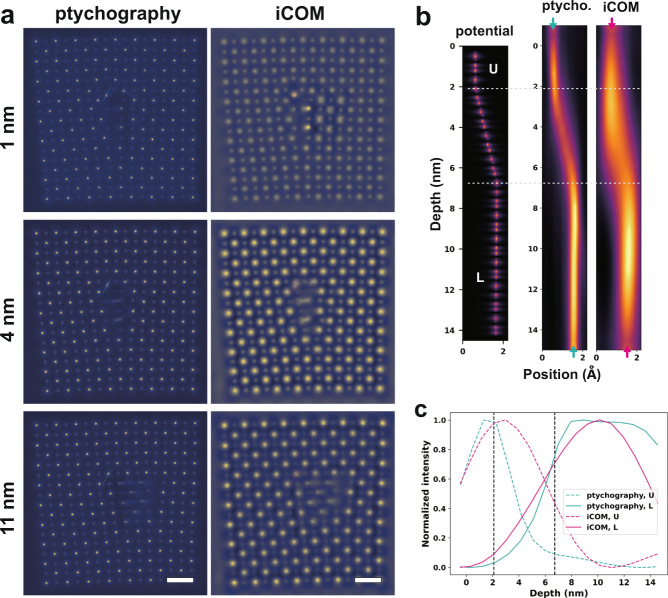
Depth sectioning via multislice ptychography and focal series iCOM. **a** Depth sections of multislice ptychography and iCOM. From top to bottom, sections at 1, 4 and 11 nm in depth. Scale bars, 5 Å. **b** Cross-sections in potential and phase images along the pathway (dashed line in **a**) linking the atomic column in the upper (U) and lower (L) sections. From left to right are the potential used to generate the simulation dataset, phase images recovered from multislice ptychography and focal series iCOM. **c** Phase profiles of the U and L sections of the atomic column shown in **b**. The vertical lines indicate the turning points along the atomic column. The smearing effect in depth is larger for iCOM than for ptychography.

Projection images are obtained by summing all depth sections recovered from multislice ptychography and focal series iCOM, which are shown in Supplementary Fig. 4. Depth sections above, at, and beneath the kink segment are displayed in Fig. 4a. As expected, due to the blurring effect of the probe, the iCOM method shows lower lateral resolution compared with multislice ptychography, resulting in lower precision in atomic displacements. Depth profiles of phases along the displacement path are given in Fig. 4b. The phase variations with depth are plotted in Fig. 4c. Comparing the results with the potential used for multislice simulation (the ground truth), multislice ptychography offers a more accurate depth profile and higher depth resolution than iCOM/iDPC. The delay of the kink onset in the depth profile of iCOM can be attributed to the channeling effect in zone axis condition. Because multislice ptychography mostly eliminates the dynamic scattering, it can provide more accurate depth variations of structure.

### Depth variations of strain and polarization

**Fig. 5:**
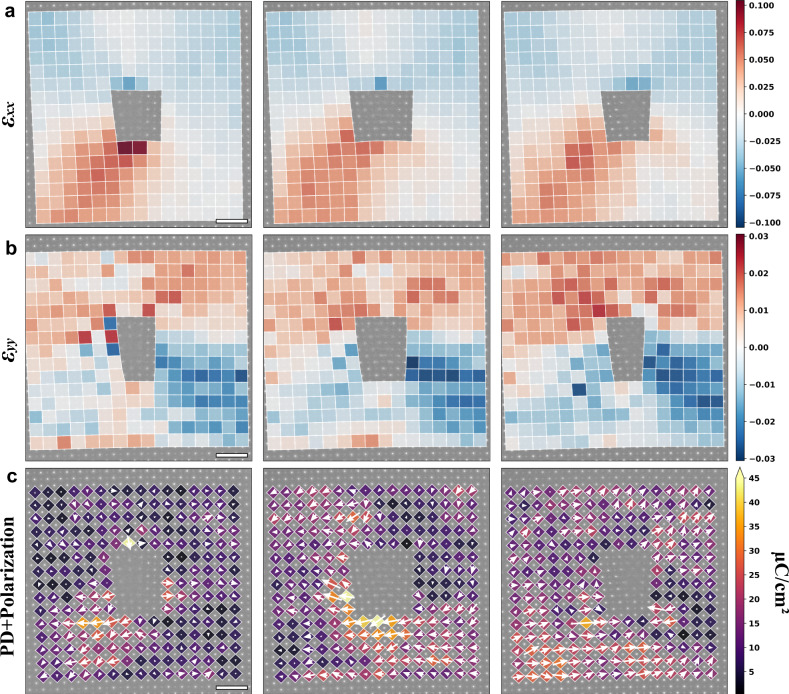
Strain and polarizations at different depth near the edge dislocation in SrTiO_3_. **a** ε_xx_ nearly parallel to the Burgers vector. **b** ε_yy_ nearly perpendicular to the Burgers vector. **c** Map of polar displacement between TiO and O columns and the corresponding polarization values. The white arrows stand for the polar displacement vectors. The quadrangles show the shapes of TiO_6_ octahedra and their color stands for the magnitude of polarizations. All the scale bars are 1 nm.

Strain induced polarization has been widely investigated in SrTiO_3_ film and related heterostructure^49,54–61^. Based on previous studies, both tensile^54,55^ and compressive strain^60,62^ can induce ferroelectricity in SrTiO_3_. Also, strain gradients at dislocations contribute to the flexoelectric polarization in SrTiO_3_^57^. The kinks discussed above can induce different strain states in depth, which offer potential driving forces to different polar displacement of Ti cations. Figure 5a, b give the strain in the *x* (*ε*_*xx*_) and y (*ε*_*yy*_) directions at different positions of the dislocation line corresponding to Fig. 3d. Relative shifts between TiO and O columns are calculated and displayed from left to right in Fig. 5c. Born effective charges are used to calculate the electric polarization (the filling color of the quadrangles in Fig. 5c) based on the measured polar displacements. However, clear correspondence between the strain state and polarization is not found. We attribute this to unattainable strain and strain gradients in the z direction and complex mechanisms for SrTiO_3_ polarization. For example, relaxorlike behavior has been observed in SrTiO_3_ thin films even no strain is applied^63^. Also, chemical factors like stoichiometry^62^ and Ti-antisite^64^ can also induce polarization, which further complicate the origin of the polarization.

## Discussions

In summary, we demonstrate the realization of sub-nanometer mapping of crystal tilt, deep sub-angstrom resolution imaging of dislocations, and depth-dependent measurements of polar displacement around dislocation cores in SrTiO_3_. Defects and concomitant crystal tilts and structure distortions are common in real materials. Electron ptychography is promising to receive broad applications to unveil the changes of structures and properties that have hidden behind conventional imaging methods.

## Methods

### Preparation of free-standing SrTiO_3_ film and TEM specimen

Thin-film heterojunctions SrTiO_3_/Sr_2_CaAl_2_O_6_ were epitaxially grown on SrTiO_3_ (001) substrates by pulsed laser deposition using a 248 nm KrF excimer laser. The Sr_2_CaAl_2_O_6_ (SAO) layer was grown at a substrate temperature *T*_g_ of 740 °C and oxygen partial pressure ${p}_{{O}_{2}}$ of 15 Pa. The SrTiO_3_ film was the grown at *T*_g_ = 710 °C and ${p}_{{O}_{2}}$ = 10 Pa. During the deposition, the energy density of laser beam focused onto the targets was 2 J cm^−2^ and 1.9 J cm^−2^ for Sr_2_CaAl_2_O_6_ and SrTiO_3_, respectively. The laser pulse rate was 3 Hz. The sample was annealed at 710 °C for 10 min while the oxygen partial pressure was maintained at 100 Pa.

To prepare TEM specimen, polymethyl-methacrylate (PMMA) was first coated onto the as-grown film, and scotch tape was attached on the surface of PMMA. Then, these three parts were immersed together in deionized water at 120 °C until the sacrificial SAO layer was completely dissolved. Next, the surface of the as-grown film was attached to the carbon film side of a TEM grid and the PMMA outside of the grid was cut. At last, the PMMA was removed by acetone vapor.

### Simulation of ptychography datasets and iCOM images

Simulations were performed using the DFTEM program (Beijing Superresolution Technology Co., Ltd.), which implements the multislice method^44,65^ and is GPU-accelerated. For simulation data, scan step sizes of ptychography and focal series iCOM are 0.3 and 0.2 Å, respectively. The simulated iCOM images were calculated from the four-dimensional (4D) datasets based on the method mentioned in Close, et al.^66^. A low pass filter removes spatial frequency below 2.5% of the band limit of probe intensity and a high pass filter removes spatial frequencies beyond 85% of the band limit of probe intensity. For 4D dataset used for multislice ptychography, the sampling interval in the reciprocal space is 0.055 Å^−1^ and each diffraction pattern contains 128 × 128 pixels, resulting in a real space pixel size of 0.14 Å. Poisson noise was added to the diffraction patterns before ptychographic and iCOM reconstruction, corresponding to a beam current of 5 pA (same as the experimental value) and acquisition time of 1 ms per diffraction pattern.

### Experimental data collection and reconstruction

A probe aberration-corrected FEI Titan Cubed Themis G2 operated at 300 kV was used to acquire the STEM images and the 4D datasets. 4D datasets were acquired with a pixel array detector EMPAD. The convergence semi-angle was set to 25 mrad. Each diffraction pattern has a dimension of 128×128 and the reciprocal pixel size is 0.055 Å^−1^. For the dataset used for reconstruction of results in the main text, the probe is under-focused about 20 nm from the optimal contrast condition of HAADF during data acquisition. Total scanning points are 256 × 256 and the step is 0.26 Å. In order to reduce the beam disturbance on the structure of dislocations, the beam current is decreased to 5 pA and the dwell time is 1 ms. Supplementary Fig. 5 shows one of the diffraction patterns. For the dataset used for reconstruction of results in Supplementary Fig. 2, the probe is under-focused about 15 nm and the scan step size is 0.52 Å. All the other acquisition parameters are the same with the first dataset.

Ptychographic reconstruction were performed using the EMPTY program (Beijing Superresolution Technology Co., Ltd.), which implements the adaptive-propagator ptychography method^42^ and is GPU-accelerated. The real space pixel size in the transmission function is 0.14 Å. Mixed-state algorithm^35,67^ with six probe modes were used, which were shown in Supplementary Fig. 6. Gradient-based drift correction method^37,68^ was used to refine scan positions. The initial and optimized scan positions are displayed in Supplementary Fig. 7.

The nominal collection angle range of the ADF, HAADF and ABF detector for images in Supplementary Fig. 1 was 25~153, 48∼200 and 8~57 mrad, respectively.

### Ferroelectric polarization

To study the effect of strain on ferroelectric properties of SrTiO_3_ film, ferroelectric polarization was calculated based on the modern theory of polarization^69,70^. According to this theory, ferroelectric spontaneous polarization can be estimated using the linear relation with Born effective charges and atomic displacements, which could be written as:

2 $$\Delta {P}_{\alpha }=\frac{e}{\Omega }\mathop{\sum}\limits_{\alpha }{Z}_{i\alpha \beta }^{*}\delta {u}_{i\beta }$$

in which, Δ*P* is the change of polarization from nonpolar state to ferroelectric state; ${Z}^{*}$ is the Born effective charge tensor of elements; $\delta u$ is atomic displacements relative to a nonpolar reference state; *e* is the elementary charge; and Ω is the volume of the unit cell. The subscript *i*, *α* and *β* are respectively the index of atoms, elements and special coordinates. The Born effective charges of SrTiO_3_ used in this work are shown in Supplementary Table 1^71^.

### Reporting summary

Further information on research design is available in the Nature Portfolio Reporting Summary linked to this article.

## Supplementary information

Supplementary Information

Description of Additional Supplementary Files

Supplementary Movie 1

Supplementary Movie 2

Lasing Reporting Summary

Reporting Summary

## Data Availability

All data that support the findings of this study have been deposited in Zenodo^72^.
