# Supplementary material for: Sub-nanometer-scale mapping of crystal orientation and depth-dependent structure of dislocation cores in SrTiO3
Source: Nat Commun. 2023 Jan 11;14:162. doi: 10.1038/s41467-023-35877-7 (PMC9834382; doi:10.1038/s41467-023-35877-7)
Supplement: Supplementary file 1 — Supplementary Information [file 41467_2023_35877_MOESM1_ESM.pdf]

Supplementary Information for

Sub-nanometer-scale mapping of crystal orientation and depth-dependent structure of dislocation cores in $SrTiO_3$

This PDF file includes:

Supplementary Figures. 1 to 9

Supplementary Tables 1 to 2

Supplementary Note 1 Transformation of tilt axis for the output crystal orientation mapping

Supplementary Note 2 Dose dependence of spatial resolution of tilt mapping

Supplementary Note 3 Theoretical depth resolution of the STEM in this work

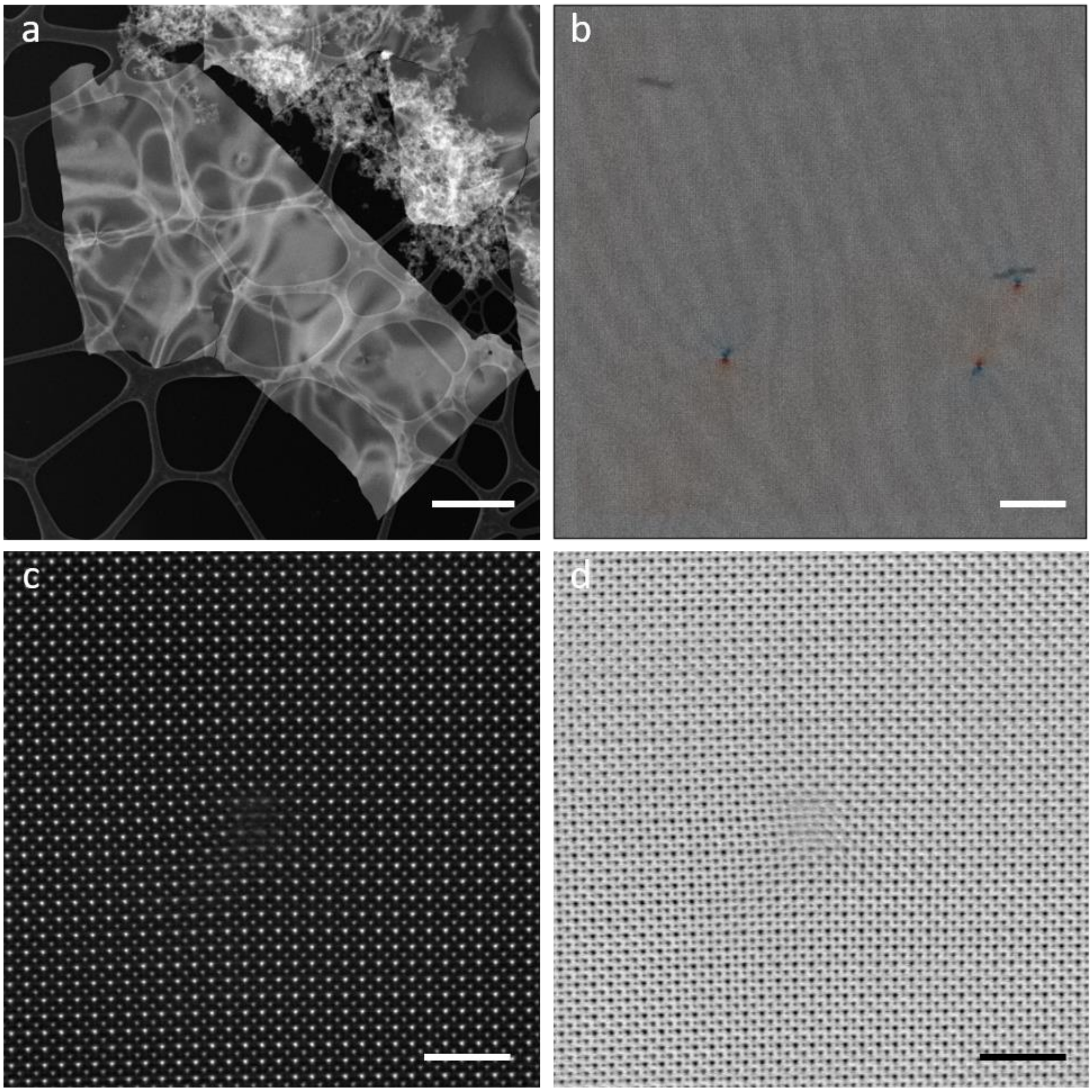

Supplementary Fig. 1. STEM images of free-standing $SrTiO_3$ film and dislocations. a, Low magnification ADF image with low inner collection angle of $SrTiO_3$ film. Scale bar, 1 μm. **b,** ADF image overlapped with ε_{xx} obtained via GPA. Dislocations can be seen from the scan moiré pattern and strain distribution. Scale bar, 15 nm. **c,** Wiener filtered HAADF image of the edge dislocation. Scale bar, 2 nm. **d,** Wiener filtered ABF image of the edge dislocation. Scale bar, 2 nm.

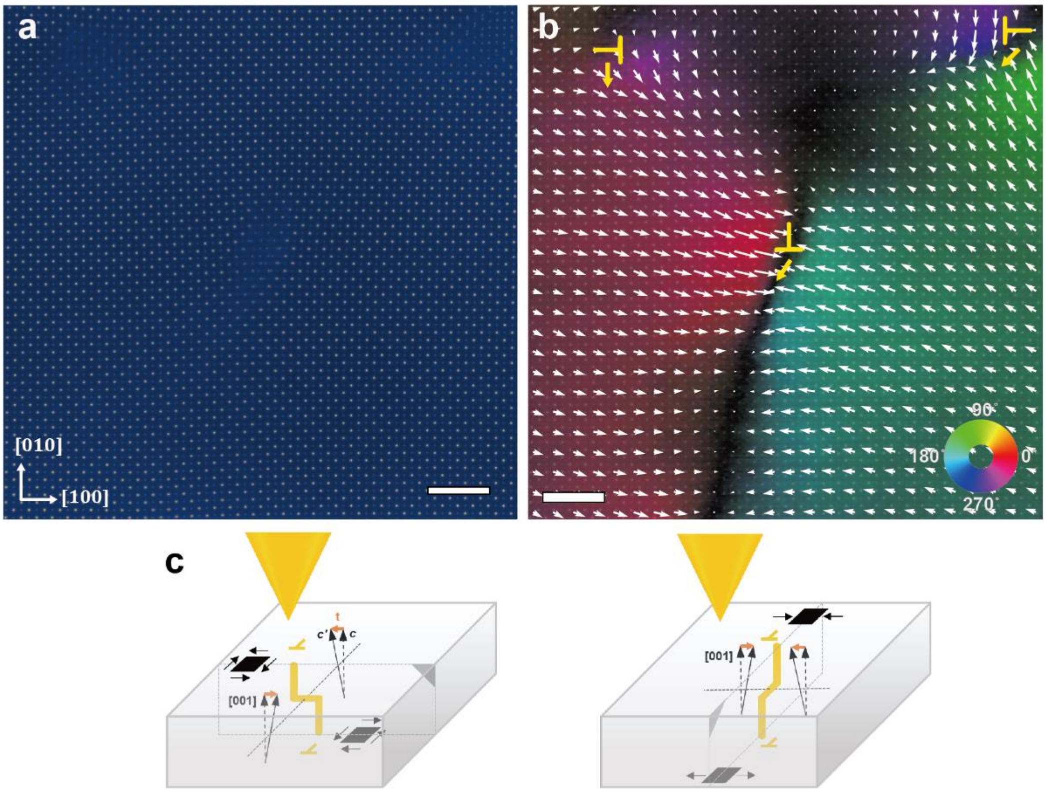

Supplementary Fig. 2. Crystal tilt mapping of multiple dislocations in $SrTiO_3$. a, Total phase image of regions containing three dislocations. **b,** Crystal tilt reconstructed in the same region of (a) with total phase image superimposed on it. The x and y components of the vector field (crystal tilts in the [100] and [010] directions) are shown in Supplementary Fig. 3e and f, respectively. The yellow arrows stand for the direction of transverse shift of the dislocation. Scale bars in (a) and (b) are 15 Å. **c,** Schematics of the relationship between transverse shift of dislocations, crystal tilt and strain states. The lattice vector $\boldsymbol{c}$ changes to $\boldsymbol{c'}$ by a tilt vector $\boldsymbol{t}$, i.e., $\boldsymbol{c'} = \boldsymbol{c} + \boldsymbol{t}$. Black squares are used to illustrate the strain state. Two modes are considered (left, torsion; right, bending). In both modes, the strain is inverted for the upper and lower part of the thin film, leading to the transverse shift of dislocations to opposite directions. The dislocation in the upper left in (b) and the one in Fig. 2 correspond to the torsion condition. The dislocations in the center and upper right of (b) correspond to a mixture of the torsion and bending.

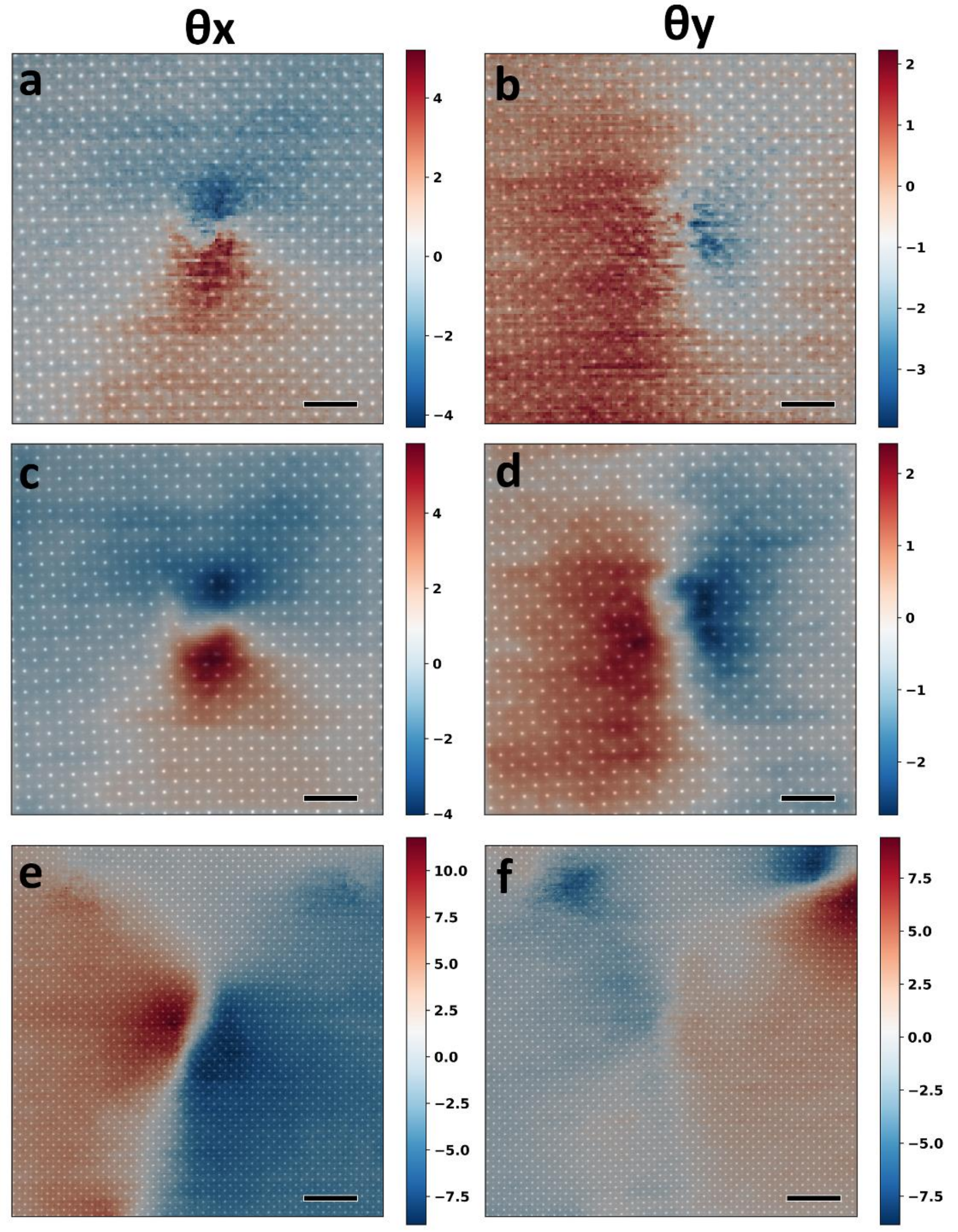

Supplementary Fig. 3. Crystal tilt distributions recovered using APP. a, b, Tilt in the [100] direction (a) and the [010] direction (b) of the region shown in **Fig. 2**a. **c, d,** The corresponding tilt with low-pass filtering during reconstructions. Tilt values are in mrad. Scale bars in (a)-(d), 1 nm. **e, f,** Tilt in the [100] direction (e) and the [010] direction (f) of the region shown in Supplementary Fig. 2. Scale bars in (e) and (f), 2 nm.

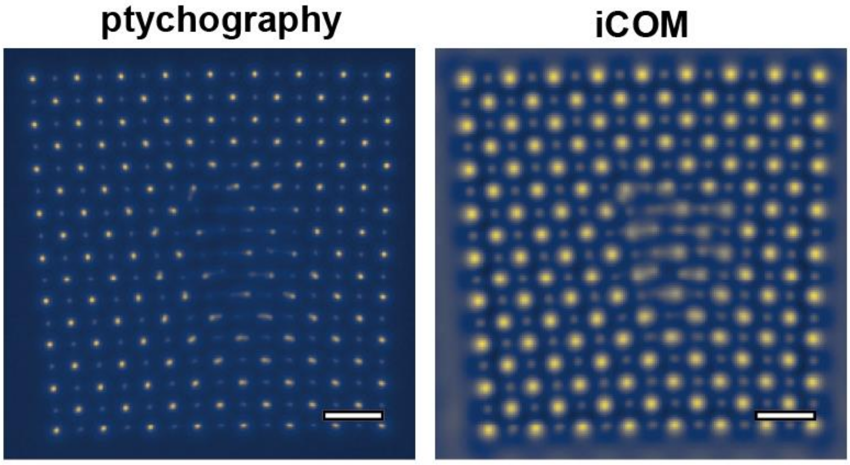

Supplementary Fig. 4. Total phase images summed over the slices for ptychography (left) and the focal series iCOM (right). Scale bar, 5 Å.

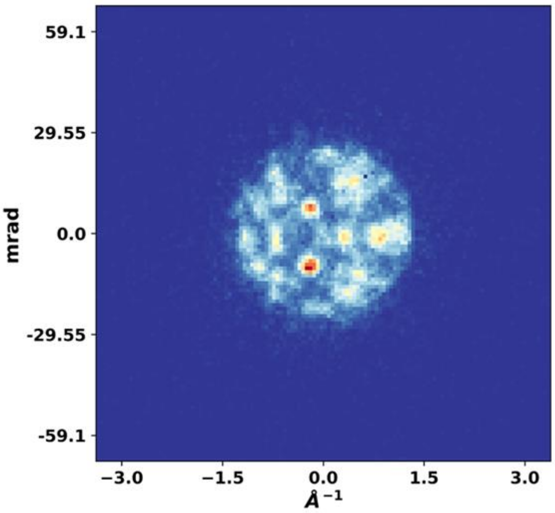

Supplementary Fig. 5. A convergent beam electron diffraction pattern under the beam current of 5 pA and acquisition time of 1 ms.

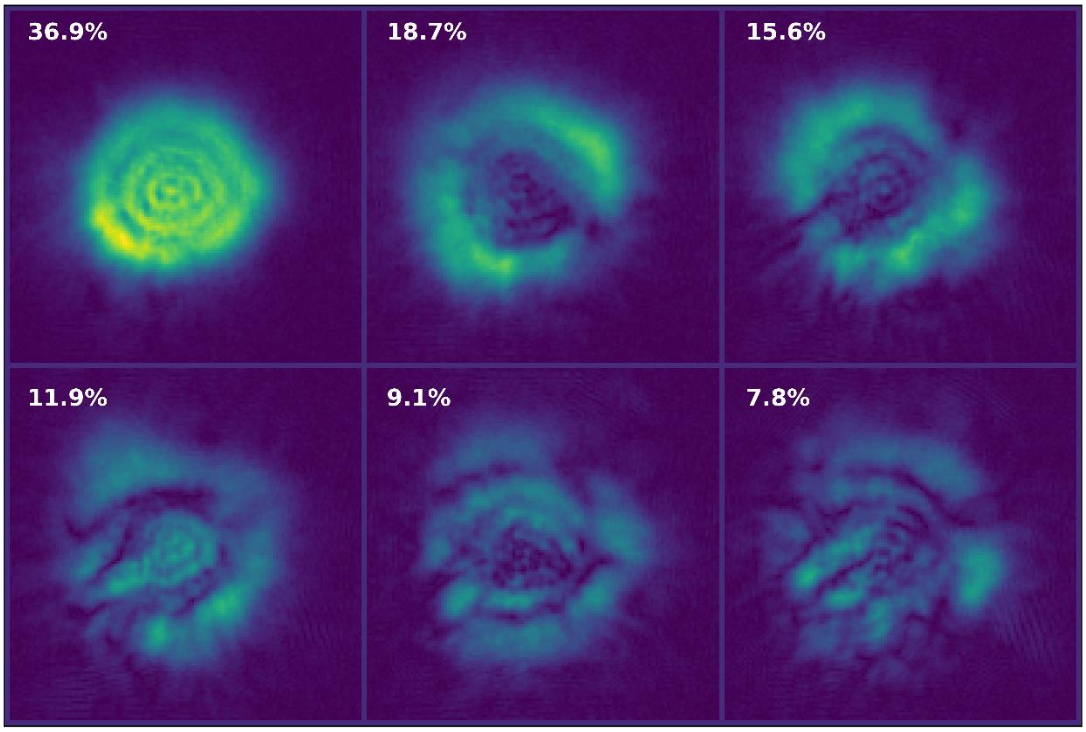

Supplementary Fig. 6. Recovered probe states in mixed-state algorithm. The ratio of the intensity of each state is labelled on the top left.

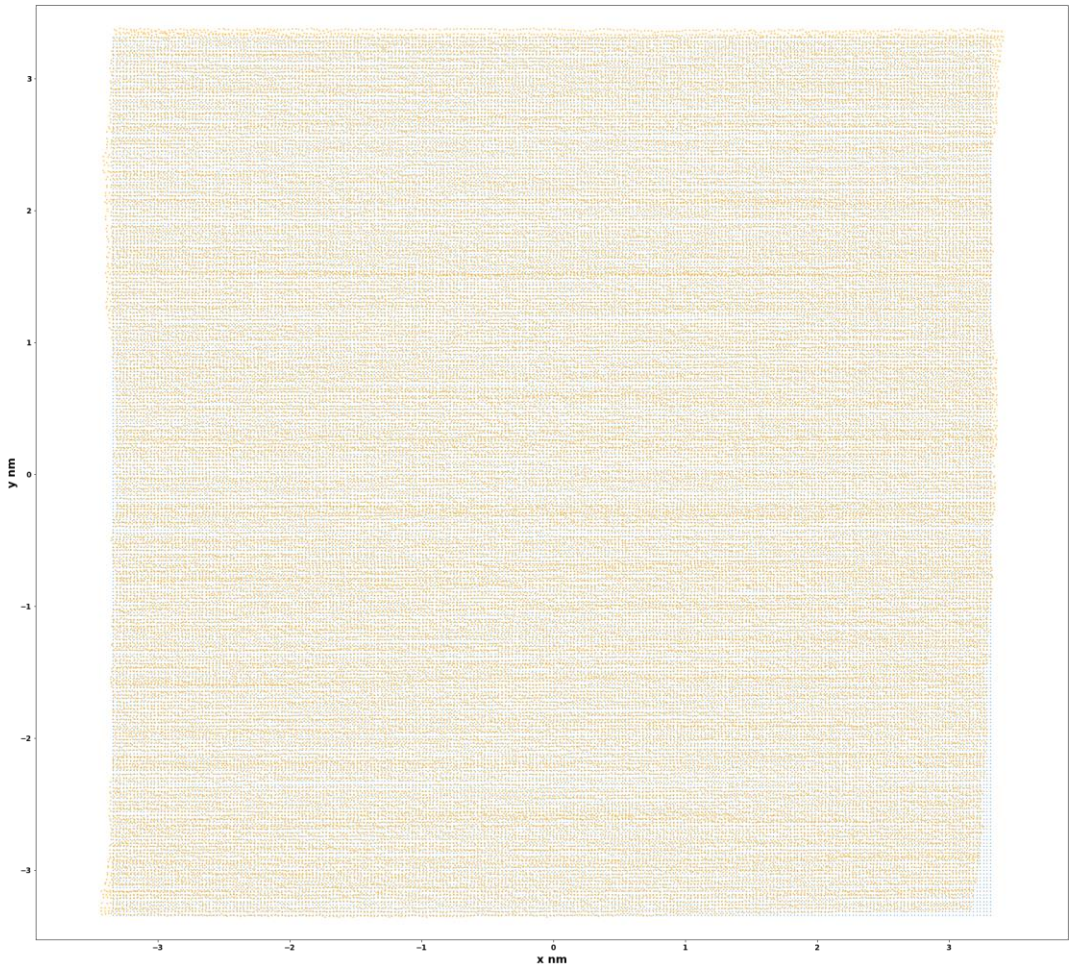

Supplementary Fig. 7. Scan positions before (blue points) and after (orange points) optimization.

Supplementary Table 1. Born effective charges of Sr, Ti and O in cubic $SrTiO_3$. The symbol // or ⊥ stands for the direction parallel to or perpendicular to the Ti-O bond respectively.

Born effective charges	Values
Z^*_{Sr}	2.4
Z^*_{Ti}	7.0
$Z^*_{\mathrm{O}\perp}$	-1.8
$Z^*_{\mathrm{O}\parallel}$	-5.8

Supplementary Note 1. Transformation of tilt axis for the output crystal orientation mapping

The tilt angles directly obtained from APP are parallel to the axes of transmission function matrix. Because of scan rotation and magnetic rotation, these axes are usually different from the crystal directions we care about. When tilt angles are small, rotation operations with respective to x and y axis are commutative and tilt can be expressed as a vector (θ_x, θ_y), where θ_x and $\theta_y$are tilt angles in the x and y directions, respectively (**Supplementary Fig. 8**c and d). As shown in **Supplementary Fig. 8**a, α is the rotation angle between the two coordinate systems. In this way, tilt angles with respect to the new axes (**Supplementary Fig. 8**e and f) are

$$\begin{pmatrix}\theta_x' \\ \theta_y'\end{pmatrix} = \begin{pmatrix}\cos\alpha & -\sin\alpha \\ \sin\alpha & \cos\alpha\end{pmatrix}\begin{pmatrix}\theta_x \\ \theta_y\end{pmatrix} \quad (1)$$

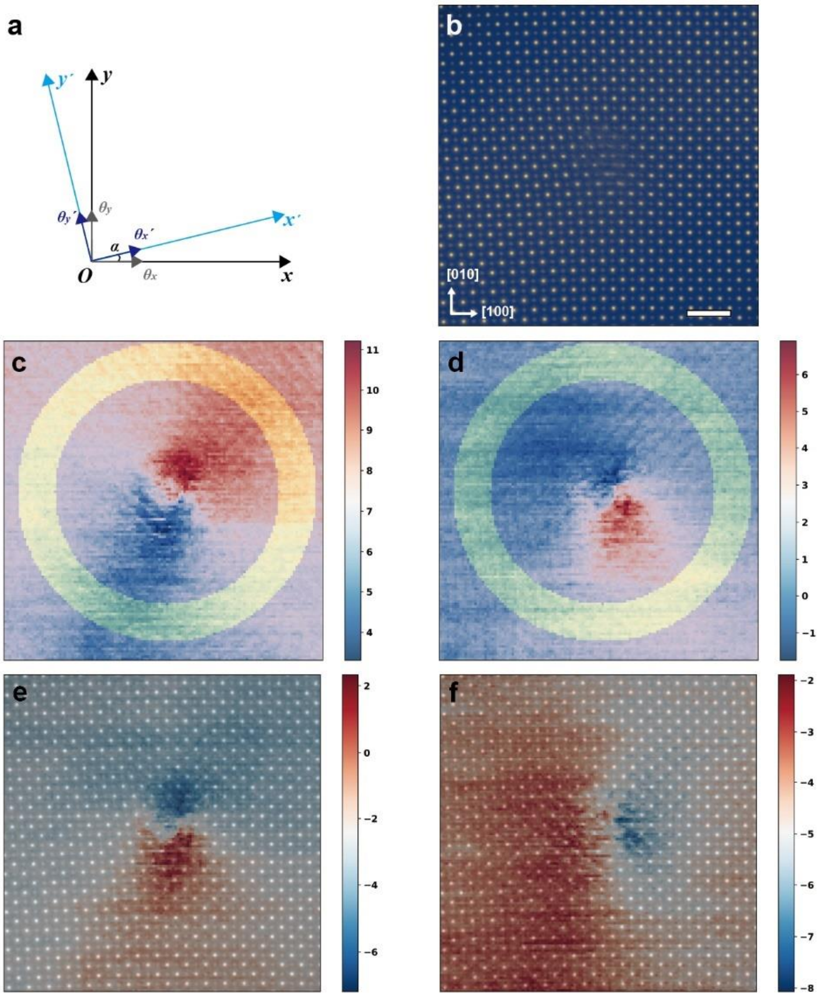

Supplementary Fig. 8. Orientation mapping before and after transformation of tilt axes. a, Schematics of tilt axes transformation. **b,** Total phase image shown in Fig. 2a. **c, d,** Output tilt angles θ_x and θ_y of APP in the x (c) and y (d) direction. Before transformation, average values in the region marked with yellow ring are subtracted from θ_x and θ_y. **e, f,** Tilt angles θ_x' (e) and θ_y' (f) after transformation.

Supplementary Note 2. Dose dependence of spatial resolution of tilt mapping

The spatial resolution of orientation mapping is investigated with an artificial $SrTiO_3$ model shown in **Supplementary Fig. 9**a. In this model, the central 5 unit-cells are tilted 6 mrad in the [010] direction. The reconstructed tilt mapping in the [010] direction are averaged along the [010] axis and the resultant tilt angle profile is displayed in **Supplementary Fig. 9**b. The recovered tilt angle profiles can be viewed as the convolution between a Gaussian function and the rectangular function representing the ground truth. The Gaussian functions are fitted and the full widths of the 80% maximum are used as resolution and displayed in **Supplementary Fig. 9**c. The ground truth of tilt values in the [100] direction is zero at every scan point and the recovered values are analyzed and displayed in **Supplementary Fig. 9**d. Standard deviations are used as error bars to demonstrate the precision of orientation mapping.

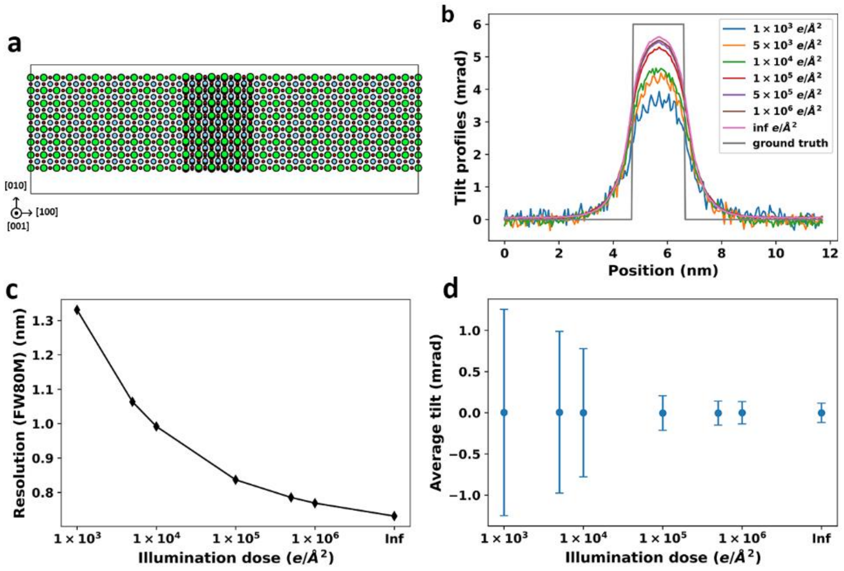

Supplementary Fig. 9. Dose dependency of resolution and precision of tilt mapping. a, $SrTiO_3$ model used for the generation of simulation 4D datasets. **b,** Tilt angle profiles obtained by averaging the tilt in the [010] direction along the [010] axis. **c,** Spatial resolution of tilt mapping as a function of electron dose. **d,** Tilt angles in the [100] direction averaged over all the scan points. Standard deviations are used as error bars.

Supplementary Note 3. Theoretical depth resolution of the STEM in this work

The depth resolution of the incoherent imaging in STEM has been obtained by numerically calculating the width of the central peak of probe intensity[1]:

$$\Delta z_0 = \frac{2.11}{\alpha_{max}}\sqrt{d_0^2 + d_c^2} \quad (2)$$

where α_{max} is the convergence semi-angle and d_0 is the diffraction-limited electron probe diameter:

$$d_0 = \frac{0.61\lambda}{\alpha_{max}} \quad (3)$$

d_c is the spread of electron probe caused by chromatic blurring:

$$d_c \approx 0.5(\sigma\alpha_{max}) \quad (4)$$

$$\sigma = \frac{C_c \Delta E}{E_0} \quad (5)$$

where σ is the defocus spread determined by chromatic aberration coefficient C_c, energy spread ΔE and beam energy E_0.

For the microscope in used in our experiment, the above parameters are listed below, giving a depth resolution of 6.5 nm.

Supplementary Table 2. Microscope parameters used to determine the depth resolution.

Microscope parameters	Values
E_0	300 keV
λ	0.0197 Å
α_{max}	25 mrad
C_c	2 mm
ΔE	0.7 eV
σ	4.7 nm
d_c	0.6 Å
d_0	0.48 Å
Δz_0	6.5 nm

Supplementary References

1. Intaraprasonk V, Xin HL, Muller DA. Analytic derivation of optimal imaging conditions for incoherent imaging in aberration-corrected electron microscopes. Ultramicroscopy 108, 1454-1466 (2008).
