# Supplementary material for: Sub-nanometer-scale mapping of crystal orientation and depth-dependent structure of dislocation cores in SrTiO3
Source: Nat Commun. 2023 Jan 11;14:162. doi: 10.1038/s41467-023-35877-7 (PMC9834382; doi:10.1038/s41467-023-35877-7)
Supplement: Supplementary file 2 — Description of Additional Supplementary Files [file 41467_2023_35877_MOESM2_ESM.pdf]

Description of Additional Supplementary Files

Supplementary Movie 1. Phases of all slices of the region shown in Fig. 2.

Supplementary Movie 2. Phases of all slices of the region shown in Supplementary Fig. 2.
