# Supplementary figures and images for: Sub-nanometer-scale mapping of crystal orientation and depth-dependent structure of dislocation cores in SrTiO3
Source: Nat Commun. 2023 Jan 11;14:162. doi: 10.1038/s41467-023-35877-7 (PMC9834382; doi:10.1038/s41467-023-35877-7)

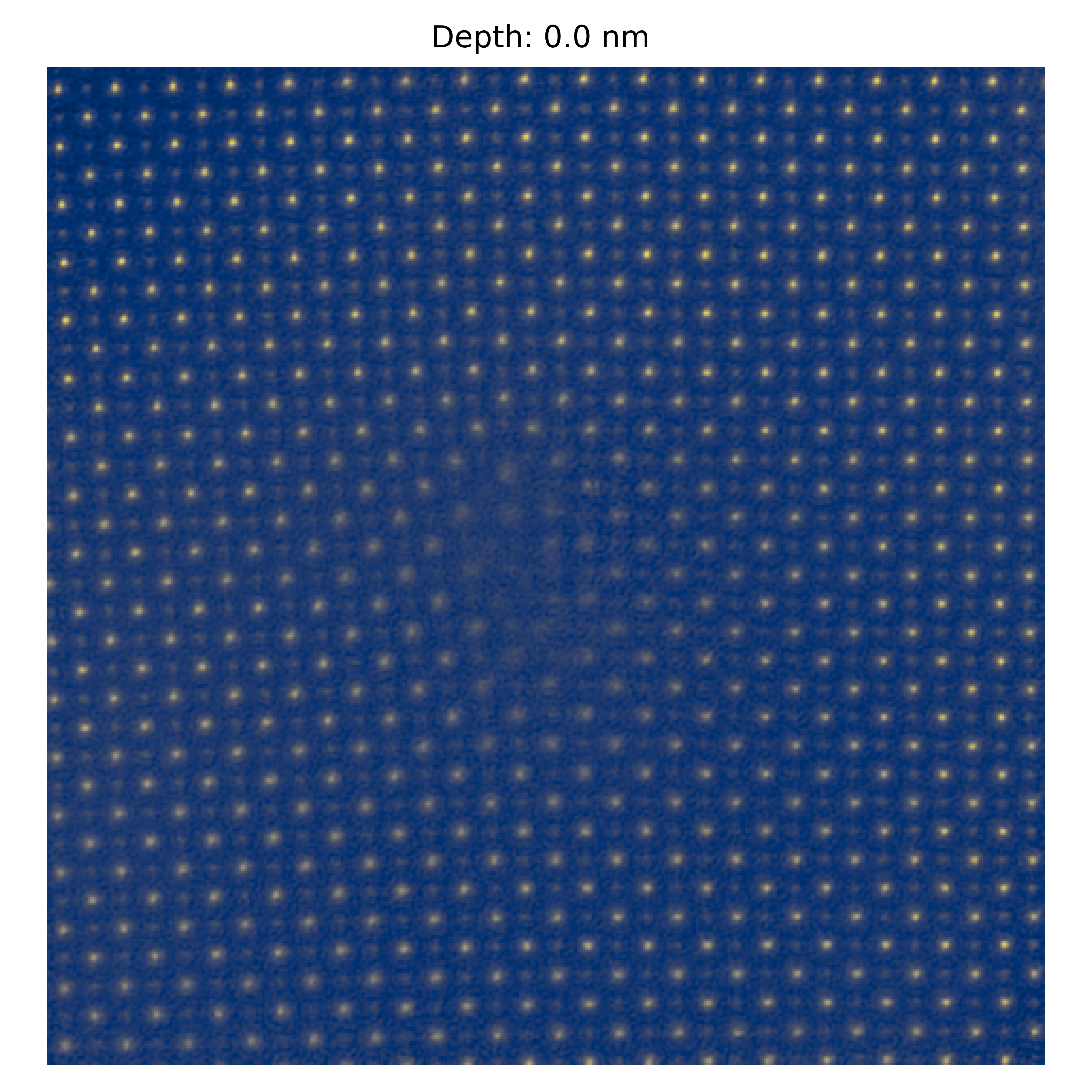

Supplement: Supplementary file 3 — Supplementary Movie 1 [file 41467_2023_35877_MOESM3_ESM.gif]
